# Comparing Learning Outcomes in Cardiopulmonary Resuscitation (CPR) and/or Automated External Defibrillator (AED) Training for Laypeople in Face-to-Face, Online, and Mixed Training Methods: An Integrative Literature Review

**DOI:** 10.7759/cureus.38489

**Published:** 2023-05-03

**Authors:** Bruno Gino, Samyah Siraj, Maria Peixoto, Andy Benson, Adam Dubrowski

**Affiliations:** 1 Emergency Medicine, Memorial University of Newfoundland, St. John's, CAN; 2 Health Sciences, Ontario Tech University, Oshawa, CAN; 3 Computer Engineering, Ontario Tech University, Oshawa, CAN; 4 Central East Prehospital Care Program (CEPCP), Lakeridge Health Hospital, Oshawa, CAN

**Keywords:** medical and healthcare, rural area, face to face teaching, virtual learning environment, drones, simulation in medical education, out of hospital cardiac arrest, emergency medical service, simulation medicine, simulation trainer

## Abstract

Cardiovascular diseases and cardiac arrest (CA) are the main causes of death worldwide. This review aims to identify publications on the learning outcomes for the use of an automated external defibrillator (AED) and/or cardiopulmonary resuscitation (CPR) to train laypeople (LP), the method of training used, the year of publication and their recommendations. We employed Miller's assessment pyramid to describe learning outcomes as knowledge, skills, and confidence. The methods of training are face-to-face, online, and mixed. The evidence found in this study will be used to support the development and validation of a simulation-based training program to teach LP to operate AEDs delivered by drones in rural and remote (R&R) locations. This article is an integrative literature review with a quantitative and qualitative research design and is composed of seven steps: research question, inclusion and exclusion criteria, search and selection of studies, the role of a second reviewer of the findings, data analysis, interpretation and discussion of the results, and finally knowledge synthesis. The results of this review demonstrate that there are no significant differences in the learning outcomes of the different training methods. Since these findings suggest good results in all methods, the development of a training program based on face-to-face, online, and mixed, especially for places with few resources such as R&R places, indicates all methods can be used as good practices to develop training programs.

## Introduction and background

Cardiac arrest (CA) can lead to an unexpected death called sudden cardiac death (SCD) [1]. Over the past 20 years, SCD is the most common cause of death outside hospitals, accounting for 25% of all global mortality [1,2]. In public places and outside hospitals, the global incidence of CA is reported to be 47.3 per 100,000 person-years in North America, 40.6 in Europe, 51.1 in Australia, and 45.9 in Asia [2-5]. Finally, in the Brazilian Amazon specifically, a descriptive and quantitative epidemiological study shows that CA is also the main cause when death occurs far from a hospital (15.05%), followed by COVID-19 (10.29%), even during the pandemic in 2020 and 2021 [6].

There is consensus in the medical literature that decreasing the response time in starting cardiopulmonary resuscitation (CPR), particularly with the use of automated external defibrillators (AED), is critical for assistance in CA [7-9]. Studies have suggested that the rapid response of the first witness is greatly important, and CPR alone, even before the arrival of an AED by the paramedic team, is independently associated with an increase in survival of up to three times longer than those situations in which the first witness does not perform any intervention on the victim or just calls 911 for help [10,11]. Additionally, mortality from CA increases by 7-10% with every minute of delay in defibrillation [12]. Therefore, shortening the time that a victim spends without defibrillation is crucial to their survival.

However, before looking for solutions on how to reduce the time victims receive defibrillation, some questions need to be answered. In out-of-hospital CA care, there are usually three people involved. First, the victim. Second, someone who witnesses the victim in CA, calls for help and initiates first aid. Third and finally, healthcare professionals who offer instructions to those who are providing first aid by remote means of communication and who will send help to the scene with AEDs. These professionals who offer instructions remotely or those who go to meet the victim in ambulances and other means of transport can be trained paramedics, nurses, or experienced emergency doctors, but this depends on the country and its protocols.

The first question is: how to reduce the delivery time of AEDs? To answer this question, we have already developed a technical report with basic life support and defibrillation (BLSD) protocols where we suggest that drones can be used for this purpose since aircraft are faster than land transport [13]. The second question: do the people who would be involved in this care have valid, evidence-based training on how to use AEDs delivered by drones? A search of scientific databases shows that there are several studies on training with AEDs for laypeople (LP) and health professionals [8,9,11,13]. On the other hand, specifically for drones that deliver AEDs, there is still a gap in the main organizations that provide medical training, such as the American Heart Association (AHA). Similar to what we have done in the past, one simulation training based on BLSD with drones was suggested but not validated by methods [13]. Finally, how do we train inexperienced healthcare professionals and LP to use AEDs delivered by drones, especially in rural and remote (R&R) settings? Answers to these questions are required to build and validate a training program that aims to teach health professionals with little training and LP to operate AEDs delivered by drones in R&R locations for CA care.

However, before building a valid training program by scientific methods for an audience located in R&R places with difficult access and few resources, a more primitive question still must be asked. This question is the research question for this literature review: between face-to-face, online, and mixed training based on SBE, what is the best combination of methods to teach LP to acquire CPR and/or AED knowledge, confidence, and skills?

## Review

Methodology

This study is an integrative literature review, a type of systematic review of the literature that uses a similar method to search for bibliographic sources, with keywords and selection criteria for the materials found [14].

The content of this review is based on the proposal published by Tranfield et al [14]. In 2023, this publication had more than 12,394 citations and the authors made a comparison between systematic reviews in medicine and the management field, proposing a method for literature reviews. For instance, he emphasizes the importance of rigor and a systematic approach in writing a literature review, which can help ensure that the review is comprehensive, reliable, and useful for researchers and practitioners.

The proposed method is divided into three stages, and each stage is divided into multiple phases [14]: Stage I: Planning the review: phase 0-identification of the need for a review; phase 1-preparation of a proposal for a review; phase 2-development of a review protocol. Stage II: Conducting a review: phase 3-identification of research papers; phase 4-selection of studies; phase 5-study quality assessment; phase 6-data extraction and monitoring progress; phase 7-data synthesis. Stage III: Reporting and dissemination: phase 8-the report and recommendations; phase 9-translating evidence into practice.

Stage I: planning the review 

The Importance of Training LP 

For this review, LP will be described as a person who does not have expert knowledge in advanced CA management and/or the use of AED (e.g., people in general, healthcare technicians, medical and nursing students in the first year of university, etc.). In the medical field specifically, several studies show that LPs who receive systematic and adequate training can triple the chances of survival of a victim with CA on-site. For example, one study showed that the development of training programs using AED for this audience, who are employees in airports and casinos, had a survival rate of 49 to 74% in victims of CA [15,16]. 

On the other hand, only teaching LP how to use AED or how to perform CPR effectively may not be enough to improve survival outcomes in locations far from hospitals, especially in R&R places. CPR alone does not ensure victim survival, but the prompt use of AEDs is critical for these scenarios [17]. However, in vast land areas with few hospitals, such as the Brazilian Amazon or Yukon in Canada, where the arrival of an ambulance would take hours or even days to arrive, seems to be one of the great challenges when investigating how to improve CA survival in R&R places.

In Canada, one study examined the feasibility of creating a drone-delivery network for AEDs in Toronto, Ontario [18]. Additionally, in 2022, a partnership between researchers from Ontario Tech University and Memorial University developed a low-cost drone called PHOENIX [13]. This aircraft can fly up to 50 kilometers and has the ability to deliver medical supplies faster than help delivered by land. These new technologies suggest that using drones for AED delivery and other time-critical medical supplies would be beneficial for survival rates and improved outcomes in places with difficult or no access to roads or ambulances.

However, before implementing a program or strategy to use drones that deliver AEDs in R&R places, it is necessary to address the training gap to make LP prepared and trained to use AEDs delivered by drones in a fully isolated environment. The importance of answering this question is highlighted by a study by researchers from the Department of Anesthesia and Intensive Care, Cork University Hospital, Ireland, which investigated the knowledge and attitudes of 142 sports club members regarding AED use [19]. The researchers found that only 20% of LP without knowledge or training in the use of these defibrillators knew how to use these devices versus 70% of people who received prior training.

Therefore, developing and validating a training program to teach LP in R&R areas how to use AEDs delivered by drones could be the first effective measure in improving health outcomes before effectively implementing a delivery system.

Simulation-Based Education (SBE) in Mixed, Face-to-Face, Online Training

In emergency disciplines, clinical skills are initially taught through theoretical classes, and later, students use simulation to develop these skills. There is strong evidence that simulation is very effective in training healthcare providers [20]. However, LPs are not trained using the same educational methods as healthcare providers to deal with CA and how to use AEDs delivered by drones in R&R places. For this reason, using similar educational methods to teach this audience to use AEDs delivered by drones in R&R places, such as the Brazilian Amazon, is promising [21-24]. 

There is a lack of studies testing the effectiveness of training programs that specifically use drones to deliver AEDs in R&R places and uncertainties about mixed training versus face-to-face and online training with these technologies. As such, the aim of this review is to compare the learning outcomes in CPR and/or AED training that use three learning methods: mixed CPR and/or AED training, face-to-face CPR and/or AED training, and finally, online CPR and/or AED training methods. 

The hypothesis is that people in R&R settings may experience greater stress because of feeling alone, so it is important to train them. With this in mind, the evidence found in this review will be used to identify the most appropriate method to develop and validate a simulation-based training program aimed at teaching individuals with no prior training in CPR and/or AED, delivered by drones, in R&R places.

Stage II: conducting a review

Objective

The objective of this review is to identify publications about the learning outcomes of the use of AED and CPR to train LP, the method of training used, the year of publication and their recommendations.

In this study, we use Miller's assessment pyramid to describe learning outcomes as knowledge, skills, and confidence [25]. The methods of training will be face-to-face, online, and mixed.

Study Design

This study is an integrative literature review with a quantitative and qualitative research design and is composed of seven steps: research question, inclusion and exclusion criteria, search and selection of studies, the role of a second reviewer of the findings, data analysis, interpretation and discussion of the results, and finally knowledge synthesis.

Step 1: Research Question

The first step was to define the research question: between face-to-face, online, and mixed training based on SBE, what is the best combination of methods to teach LP to acquire CPR and/or AED knowledge, confidence, and skills?

Step 2: Inclusion and Exclusion Criteria

As suggested by Tranfield et al. [14], the second step is to establish the inclusion and exclusion criteria.

Inclusion criteria: Articles written in English, free full-text, clinical trial, meta-analysis, randomized controlled trial, systematic review, CPR and/or AED training studies for LP through SBE, publication date from 2000/1/1 to 2020/12/31, and surveys that compared two or more CPR and/or AED training methodology groups.

Exclusion criteria: Articles published in languages other than English, duplicate studies, articles with access links that were unavailable at the time of collection, articles with a publication period prior to January 1st, 2000, and after December 31st, 2020, and studies that did not correspond to the objectives of the study. 

Step 3: Search and Selection of Studies

The third step was characterized by the search and selection of articles in the Google Scholar and PubMed databases. However, as there is a lack of studies focused on training programs with LP that use AEDs delivered by drones, the PICO framework was used to address the research question by identifying articles that discuss CPR and/or AED training. The four stages of the PICO framework include (P) population, (I) intervention, (C) comparison, and (O) outcome [26].

Population (P): The first strategy was to identify the population in which this review would carry out the research: LP is not trained in the use of AED. 

Intervention (I): The second strategy was to identify the intervention: the standard AED method of training with mannequins, smartphones, virtual reality (VR), and augmented reality (AR). 

Comparison (C): The third strategy was to identify the comparison: compare the face-to-face learning outcomes of teaching CPR and/or AED with mannequins to alternative AED methods of training (online and/or mixed). 

Outcome (O): Finally, the fourth strategy was to identify the outcomes of the training, such as AED knowledge, quality, and skill performance, for each of the methods of training.

Through the databases, articles were searched between January 1st, 2000, and December 31st, 2020. Medical subject headings (MeSH) were searched using the Boolean operators “OR/AND.” The following terms were used for the search strategy: (“automated external defibrillator” OR “AED”) AND (“hands-only CPR” OR “cardiopulmonary resuscitation” OR “CPR”) AND (“teaching methodologies” OR “training methods”) AND (“simulation”) AND (“bystanders” OR “laypersons”). A total of 108 studies were selected to be screened.

For PubMed, the following filters were applied: Free full-text, clinical trial, meta-analysis, and English, from January 1st, 2000 to December 31st, 2020. A total of three articles were discovered.

For Google Scholar, the following filters were applied: English, from January 1st, 2000 to December 31st, 2020, sorted by relevance, any type and include citations. A total of 105 articles were discovered.

To select the studies for this literature review, three screening stages were conducted by one researcher.

Screening stage 1: The titles and abstracts of the articles were first reviewed, and those that met the inclusion criteria were chosen for the full-text review.

Screening stage 2: The articles were reviewed by reading the full text, and 14 articles were selected for this literature review.

Screening stage 3: In the 14 articles selected, only those with study designs involving LP as a target audience, clinical trials, meta-analyses, randomized controlled trials, or systematic reviews were considered.

After searches in Google Scholar and PubMed, a total of 108 relevant articles were found. Of the 108 articles, one duplicate was identified, five were not accessible as the webpage did not open, and 13 were written in a language other than English and therefore were all excluded. A total of 89 potentially relevant articles were reviewed. After reading the titles and abstracts, 73 articles did not meet the eligibility criteria for the study designs and were excluded. Finally, 16 articles went through the full-text screening process. Two articles studied training programs with children and were excluded. In the end, 14 articles were chosen for this review.

**Figure 1:**
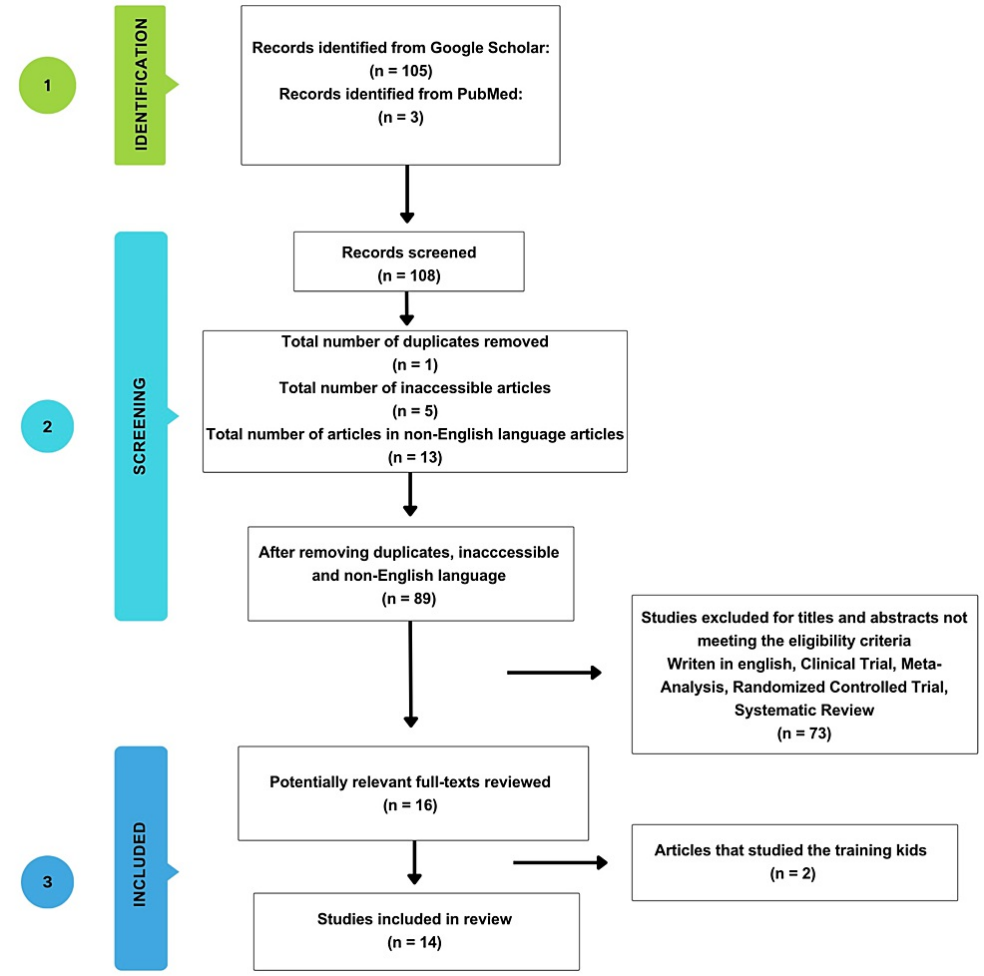
A flow chart of the process of selecting articles.

Microsoft Excel (Microsoft Corporation, New York, USA) was used to organize the data, and a flow chart detailing the process of selecting the articles was created (see Figure 1).

Step 4: The Role of a Second Reviewer of the Findings

Two researchers (BG and SS) reviewed the 14 studies. The purpose of this was to ensure the validity of the findings and to check the results. For this review, no studies were excluded based on the quality assessment, and the researchers made this decision since this synthesis of literature aims to include the search for all articles relevant to our review question. 

Step 5: Data Analysis

The fifth step was characterized by data analysis. The data was grouped according to the objectives of this review. For example, articles in which authors chose people with little or no knowledge of AEDs or CPR, who analyzed the quality of learning outcomes in face-to-face, online, and mixed training, year of publication between 2000 and 2020 and their training recommendations.

Step 6: Interpretation and Discussion of the Results

The sixth step was the interpretation and discussion of the results indicated in the literature. In this stage, after selecting the articles, the data were recorded in an Excel spreadsheet and organized into publication year, country, study design, sample size, target group, outcome measures, and finally training method.

Step 7: Knowledge Synthesis

Finally, the seventh step was characterized by an elaboration of the knowledge synthesis through the results of this review. After completing each step, a final sample of 14 publications was analyzed according to the objective of this review. The articles have been divided and organized based on: year of publication, country, study design, sample size, target group, learning outcomes, and method of training. The results are presented in Table 1.

**Table 1:** Details of the selected articles. LP: laypeople, CPR: cardiopulmonary resuscitation, BLS: basic life support, BLSD: basic life support and defibrillation, AED: automated external defibrillator, DVD: digital video disc, VR: virtual reality, AR: augmented reality.

Year and first author	Country	Study design	Sample size	Target group	Learning outcomes	Method of training	Keynotes
2019, Alexander et al. [27]	USA	Retrospective study	9022	LP	Confidence	Face-to-face	The study concludes that individuals are more likely to be currently CPR-trained in states with mandatory CPR training upon high school graduation.
2013, Creutzfeldt et al. [28]	USA and Sweden	A prospective international collaborative study	36	LP	Confidence/knowledge	Online	The study concludes that using the online method of team CPR training is feasible and reliable for this high school LP group (Sweden and US). A high level of appreciation was reported among these participants.
2019, Hansen et al. [29]	Denmark	Randomized controlled trial	247	LP	Confidence/skills/knowledge	Mixed	The study concluded that there was no difference in pass rate when comparing a demo with a lecture. On the other hand, the lecture group was slightly faster in starting the BLS when prompted, and most participants preferred a demonstration as an introduction.
2007, Harve et al. [30]	Finland	Randomized controlled trial	44	LP	Skills	Mixed	The study concludes that the quality of dispatcher-assisted CPR is poor. On the other hand, the dispatcher's assistance in defibrillation by a layperson not trained in CPR and to use an AED is feasible. It does not compromise the performance of the intervention.
2018, Hatakeyama et al. [31]	Japan	Randomized controlled study	61	LP	Skills	Mixed	The study concludes that the analyzed training test showed that the AED delivery time was shortened by the newly developed smartphone app for the layman to ask nearby rescuers to find and bring an AED to the scene of cardiac arrest.
2018, Hsieh et al. [32]	Taiwan	Randomized controlled trial	96	LP	Skills/knowledge	Mixed	The study concluded that LPs with a three-month retraining interval had the highest pass rate when performing conventional CPR. On the other hand, a six-month retraining interval can be considered, but with lower learning outcomes.
2005, Isbye et al. [33]	Denmark	Randomized controlled trial	238	LP	Skills	Mixed	The study concludes that when LP is evaluated after three months, a 24-minute DVD-based instruction plus subsequent self-training in BLS appears equally effective compared to a six-hour BLS course and is, therefore, more efficient.
2019, Leary et al. [34]	USA	Randomized controlled trial	105	LP	Knowledge/confidence	Online	The study concludes that the use of VR significantly increased the likelihood that an LP would call 911 and request an AED.
2017, Zinckernagel et al. [35]	Denmark	Qualitative study based on interviews	25	LP	Confidence	Face-to-face	The study concludes that LPs having previous training and even a little knowledge about defibrillators were crucial for their perception of training.
2016, Vetter et al. [36]	USA	Prospective trial	412	LP	Skills	Face-to-face	The study concludes that LPs who underwent creative and innovative methods of teaching and learning resuscitation skills showed excellent application of these skills in a simulated code with remarkable retention of psychomotor skills.
2019, Hsu et al. [37]	Taiwan	Pilot study	95	LP	Knowledge	Face-to-Face	The study concludes that teaching CPR first in a standardized face-to-face education program improved participants' ability to recognize cardiac arrest and activate the emergency response system.
2020, Ingrassia et al. [38]	Italy	Usability study	26	LP	Knowledge	Mixed	The study concludes that the BLSD AR system is a viable and acceptable tool for LP training.
2015, Hernández-Padilla et al. [39]	United Kingdom	Cluster randomized trial	177	LP	Confidence/skills/knowledge	Face-to-face	The study concludes that using a learner-directed strategy to retrain skills resulted in a greater proportion of LPs achieving and retaining competence in AED use at three months compared to an instructor-led strategy.
2008, Hamasu et al. [40]	Japan	Questionnaire and surveys	259	LP	Knowledge	Face-to-face	The study concluded that LP who showed a willingness to perform and learn to use AED increased after face-to-face training.

Stage III: reporting and dissemination

Findings

The population of the selected studies was LP, such as high school students, teachers, and medical and/or nursing students in their early school years who had yet to receive training in CPR and/or AEDs. 

The studies selected for this review evaluated three main learning outcomes after training participants in CPR and/or AEDs using mixed, face-to-face, or online methods of training. The learning outcomes were grouped into the CPR and/or AEDs training in confidence, skills, and knowledge of participants. The face-to-face method of training includes the use of mannequins and face-to-face demonstrations. Online methods of training involves technologies, including instructional videos, serious games, virtual reality (VR), and augmented reality (AR). Those that used both face-to-face and online methods of training were referred to as mixed methods of training.

Comparing Learning Outcomes in Confidence

Six studies demonstrated improvement in confidence during face-to-face, online, and mixed CPR, and/or AED training [27-29,34,35,39]. Self-instruction videos, computers, online and face-to-face lectures with periodic retraining, and VR glasses and smartphone applications were explored to test the effectiveness of these methods of training on participant confidence. One face-to-face training study by Alexander et al. [27] stated that people living in states in the U.S. that require mandatory CPR courses to be completed for high school graduation were more likely to self-indicate that they were trained and felt confident in providing help if they witness a CA victim than those who lived in states where this course was not mandatory. Additionally, an online training study by Creutzfeldt et al. [28] involving teenagers showed a high level of appreciation reported among the participants, and their confidence significantly increased after the course. Another mixed-training study by Hansen et al. [29] also demonstrated that participants felt more confident after being introduced to AED devices with face-to-face practical demonstrations of mannequins. However, in this same study, of those who simply heard about these devices in online lectures, 91% indicated that they would feel more comfortable with face-to-face training. In an online study by Leary et al. [34] using VR, it was shown that 68% of participants felt that they would be able to identify a CA, and 52% declared themselves able to save lives at the end of the course. In addition, Zinckernagel et al. [35] showed that the simple fact of knowing and having manual contact with an AED and also, recognizing that this device does not cause harm to the operator, made participants accept being trained and feel more confident during training. Finally, Hernández-Padilla et al. [39] applied questionnaires at the end of the course with small groups showing that the participants felt less anxious when operating an AED [39]. None of the studies analyzed, which used mixed, face-to-face, and online training methods, showed low results in confidence regarding learning outcomes from Miller's assessment pyramid. 

Comparing Learning Outcomes in Skills

In terms of skills acquired in face-to-face, online, and mixed training, seven studies were able to use different teaching strategies [29-33,36,39]. Breaks between courses and reviewing information between these breaks, as well as technologies and creative ways of teaching, were explored to test the effectiveness of these methods of training to apply assessments to evaluate the skills of the participants.

In a study by Hsieh et al. [32] involving mixed training in basic life support (BLS), the participants who had both online and face-to-face training were asked to demonstrate their skills at the end of the course. However, the results showed that face-to-face training was not superior to online. On the other hand, the findings indicated that in terms of time between refresher training, the results showed that LP with a three-month retraining interval had a high success rate in using an AED correctly. However, an interval of six months can also be considered for good quality training for both training methods of training. In addition, Isbye et al. [33] showed that a quick 24-minute online training for LP was as effective as a six-hour course when teaching face-to-face CPR training. Three other studies on mixed and face-to-face training, conducted by Hatakeyama et al. [31], Vetter et al. [36], and Hernández-Padilla et al. [39], indicated that software utilizing creative and innovative methods to teach resuscitation skills yielded excellent results in simulated environments.

A mixed study by Harve et al. [30] was performed with 95 LPs, which were divided into group 1 and group 2. The researchers showed that group 1 was taught first to recognize CA and how to start CPR, and group 2 to use AED only. The results showed that group 1 acquired greater skill performance than group 2. Finally, Hansen et al. [29] concluded that both face-to-face and online methods improved skills. However, the face-to-face lecture group was faster at initiating BLS protocols. None of the mixed and face-to-face training studies by Hansen et al. [29] and Harve et al. [30] demonstrated poor learning outcomes in skills. However, in these mixed training studies, the participants that trained face-to-face showed better results. No study that conducted exclusively online training evaluated skill learning outcomes.

Comparing Learning Outcomes in Knowledge

In terms of knowledge tested during face-to-face, online, and mixed training, eight studies executed different methods of training to apply learning outcomes for participants [28,29,32,34,37-40]. In two studies by Leary et al. [34] and Ingrassia et al. [38] comparing online and mixed training using new technologies such as AR and VR to face-to-face training, the knowledge that the participants acquired by understanding their safety and attitude to ask for an AED as soon as possible increased with the strategy of using online methods such as VR in self-training and AR in recognizing the environment. This effective knowledge retention method has also been demonstrated through both face-to-face lectures and online methods by Hansen et al. [29]. On the other hand, specifically in knowledge tests, one online method by Leary et al. [34] demonstrated that LP interacting with new online technologies identified that the public was more active, focused, and prone to respond faster and also more confident in demonstrating solid knowledge retention about the first steps of BLS protocols. Regarding VR, Creutzfeldt et al. [28] suggested that serious games-based training is a feasible approach to teaching students and teachers how to use an AED to adequately respond to an emergency with good acceptance and good quality results. However, it seems that using these technologies requires a certain level of prior knowledge for both students and teachers, but not training in BLS techniques. The retraining time was also crucial to assess knowledge retention, as demonstrated by face-to-face and mixed training, as demonstrated by Hsieh et al. [32], Hsu et al. [37], and Hernández-Padilla et al [39]. Hsieh et al. [32] showed that participants who retrained every three months showed better results than those who were retrained every six months. On the other hand, Hsu et al. [37] conducted a pre- and post-test immediately and a post-test six months later, in teaching CPR first. The results of this study show that this attitude improves the knowledge results evaluated for both tests in face-to-face training. In addition, a face-to-face training study by Hernández-Padilla et al. [39] showed that four hours of training is enough to have good knowledge results.

Finally, Hamasu et al. [40] demonstrated that face-to-face BLS training, where LP learned to use AED in small groups, significantly increased knowledge retention by recognizing the first steps of BLS protocols such as calling for help and quickly initiating CPR. None of the mixed, face-to-face, and online training studies showed low results when carrying out knowledge assessments. On the other hand, studies that evaluated training and retraining time showed that the minimum time required to obtain good results is four hours per session, and the retraining should be performed at a maximum of every three months to achieve excellent results.

Results

The 14 articles analyzed in this review compared learning outcomes for face-to-face, online, and mixed CPR and/or AEDs training in confidence (42.9%), skills (50%), and knowledge (57.1%). The most prevalent years of publication were between 2015 and 2020 (71.4%). The types of study designs were respectively the “randomized controlled trial” (42.8%), “Retrospective study” (6.6%), “A prospective international collaborative study” (6.6%), “Qualitative study based on interviews” (6.6%), “Prospective trial” (6.6%), “Questionnaire and surveys” (6.6%), Pilot study (6.6%), “Usability Study” (6.6%), and “Cluster Randomized trial” (6.6%). The sample sizes were >100 and <9022 (46.7%) and >13 and <100 (53.3%). The target groups were LP (100%).

Discussion

This review aimed to answer the following research question: "Between face-to-face, online, and mixed training based on SBE, what is the best combination of methods to teach LP to acquire CPR and/or AED knowledge, confidence, and skills?" In general, the findings suggest that there were no training methods superior to others when comparing, face-to-face, online, and mixed training. In addition, although the skills were not analyzed in studies that only used an online training method, such as the ability to perform specific tasks or procedures, the studies reviewed showed that all training methods were equivalent in their effect on confidence, skills, and knowledge.

In terms of confidence, all studies with face-to-face and mixed training showed that these methods were equivalent in the final result of learning outcomes. For instance, Alexander et al. [27] and Hansen et al. [29] showed that LP who had face-to-face experiences declared themselves highly confident in using the AED and had both excellent learning outcomes. In addition, Alexander et al. [27] and Zinckernagel et al. [35] both suggested that participants living in U.S. states where face-to-face training was mandatory, and those who received instructions on AED safety, showed great self-declaration of feeling able to help people with CA. Furthermore, Hernández-Padilla et al. [39] demonstrated that constant training led participants to report high confidence levels. Additionally, Creutzfeldt et al. [28] reported that participants who received online training methods experienced positive growth in confidence and concentration during the evaluation. Also, Leary et al. [34] demonstrated that 52% felt they were able to handle CA when training with VR or apps, and 68% felt very capable of recognizing this condition. In summary, when we analyze the studies that used face-to-face, online, and mixed methods for learning outcomes in confidence, this review suggests that all three methods are equally good for teaching. For skills, the studies reviewed also demonstrated good learning outcomes in all three methods. For instance, Hansen et al. [29] and Hatakeyama et al. [31] concluded that face-to-face and mixed methods, including one carried out by lectures, smartphones, and virtual reality improve skills. In addition, participants of the study by Hansen et al. [29] and Harve et al. [30] in mixed training who had online lectures and instructions by telephone and prior training were faster to start BLS. On the other hand, in one mixed-method training when participants had the opportunity to update their skills, the mixed training showed excellent retention when analyzing manual skills [33]. Further, when groups were trained every three months, Hsieh et al. [32] concluded that those who had face-to-face training showed excellent skills. Additionally, when Vetter et al. [36] evaluated skills in face-to-face training, they discovered that participants who underwent retraining every three months also achieved good results. In terms of knowledge acquisition as well as skills and confidence, all reviewed studies showed good learning outcomes in face-to-face, online, and mixed methods. Hernández-Padilla et al. [39] and Hamasu et al. [40] verified that participants were tested before and after face-to-face training, and high retention was verified to recognize CA, operate AED and follow BLS protocols. On the other hand, it was found by Hsu et al. [37] that the retraining time cannot exceed six months for participants to demonstrate as good knowledge retention as retraining after three months. Finally, of all the studies that evaluated the retention of knowledge, only one face-to-face training by Hamasu et al. [40] demonstrated a real impact and drastically changed the assessment of knowledge. This is because participants received training in small groups, which led to a small proportion of participants showing an improvement in their willingness to undertake the training. In addition, small groups had good results in knowledge about safety and efficiency when performing BLS protocols, and these results increased from 13% to 77% after the program. Finally, there was no study that used face-to-face, online, or mixed methods to evaluate knowledge acquisition with negative results.

The results of this review demonstrated that there are no significant differences in the learning outcomes of the different training methods. Since these findings suggest good learning assessment in all methods, the development of a training program based on face-to-face, online, and mixed, especially for places with few resources such as R&R places, indicates all methods can be used as good practices to develop training programs. For example, there are health professionals and students in remote locations, and although these people have few resources, they have access to the Internet. In this specific case, the online methodology would be ideal. On the other hand, those who live in urban areas can benefit from face-to-face teaching and this course can be adapted to a mixed or even online without prejudice if there is a new outbreak of the COVID-19 disease in the future. Additionally to this, the researchers of this review published an article in 2022 [13] that described a face-to-face simulation scenario to teach health professionals to deal with CA by using AEDs delivered by drones in R&R locations. 

Finally, the results of this review will be used to develop and validate a training program based on scientific evidence. Further, the evidence found in this paper will provide support to researchers to choose any method and obtain good learning outcomes in a course to teach people with little or no training in CA to operate drone-delivered AEDs at R&R places safely and effectively.

Limitations of the study

Four limitations were identified for this literature review. First, no selected studies have tested the skills of online training. Second, none of the participants had the chance to participate in a real CA, and therefore, it is not possible to know how they would perform when dealing with this event in the real world. Third, none of the 14 studies measured the impact of different SBE methodologies at the population level. Finally, all the studies were carried out in developed countries with high-cost training programs. For this reason, adopting the same scenario effectively to developing countries and in R&R areas such as Amazon, where these technologies are not accessible, makes implementation difficult or even impossible.

## Conclusions

This literature review studied CPR and/or AED face-to-face, online, and mixed training methods, all based on SBE, and assessed the confidence, skills, and knowledge of LP. Positive results were observed in all studies involving face-to-face training and using methods such as demonstrations, lectures, videos, computers, new technologies, such as serious games, smartphones, AR, and VR. It was observed that studies using online training methods did not assess skills, and this may be because the participants were far from the researchers. In addition, it was also observed that the best time to retrain participants in mixed, face-to-face, and online programs is retraining every three months or no longer than six months. After twelve months, the evaluation results in confidence, skills, and knowledge decreased drastically.

Due to the promising results observed in all teaching methods, this review raises evidence that any mixed, face-to-face, and online training methods compared with each other and evaluated separately, can be effectively applied to develop and validate a training program with good results. Specifically, to teach LP, such as healthcare technicians in the heart of the Amazon or in very northern places in Canada and other R&R areas, how to operate AEDs delivered by drones, all methods have the potential to develop and validate training programs based on SBE.

Finally, this evidence can be used as an incentive to develop uniformity in methodology when implementing a training program, which will make the comparison of teaching techniques based on alternative simulations acceptable at the time of implementation. In addition, the flexibility that training centers may have regarding the teaching method, especially in times of the coronavirus pandemic when distance learning may be mandatory and there is no possibility of carrying out face-to-face training, this study suggests that regardless of the method chosen, the results are equally acceptable.
